# Clinical and epidemiological characteristics of the 2025 dengue outbreak in Bangladesh

**DOI:** 10.1371/journal.pntd.0014661

**Published:** 2026-08-26

**Authors:** Tamanna Tabassum, Sadia Zaman, Md Mohiuddin Khan, Mohammad Jahid Hasan, Md. Shahabul Huda Chowdhury, Muna Islam, Sadia Islam, Jannatul Fardous, Mehraj Islam, Md. Atiquel Islam Chowdhury, Madhabi Karmaker, Mohiuddin Sharif, Quazi Tarikul Islam

**Affiliations:** 1 Pi Research & Development Center, Dhaka, Bangladesh; 2 Curtin University, Western Australia, Bentley, Australia; 3 Department of Medicine, Mymensingh Medical College Hospital, Mymensingh, Bangladesh; 4 Tropical Disease and Health Research Center, Dhaka, Bangladesh; 5 Department of Medicine, Dhaka Medical College, Dhaka, Bangladesh; 6 Department of Medicine, Chattogram Medical College Hospital, Chattogram, Bangladesh; 7 Department of Medicine, Delta Medical College, Dhaka, Bangladesh; 8 National Institute of Mental Health (NIMH), Psychiatry, Dhaka, Bangladesh; 9 Department of Medicine, Popular Medical College Hospital, Dhaka, Bangladesh; 10 Department of Medicine, Southern Medical College, Chattogram, Bangladesh; 11 Department of Medicine, Dhaka Medical College Hospital, Dhaka, Bangladesh; 12 Dhaka Medical College Hospital, Dhaka, Bangladesh; 13 Popular Medical College, Dhanmondi, Dhaka, Bangladesh; Center for Disease Control and Prevention, UNITED STATES OF AMERICA

## Abstract

**Background:**

This study aimed to describe the clinical and epidemiological characteristics of patients with dengue fever during the 2025 outbreak in Bangladesh.

**Methods:**

This multicenter observational study was conducted in four tertiary care hospitals in Bangladesh between June and October 2025. Hospitalized adult patients with laboratory-confirmed dengue infection, defined by a positive NS1 antigen or dengue IgM test, were consecutively enrolled. Demographic characteristics, clinical manifestations, and laboratory parameters were collected through face-to-face interviews using a pretested semi-structured case record form. Physical examination findings and other relevant clinical information were extracted from medical records at the time of hospital admission. Dengue severity was classified according to the revised World Health Organization (WHO) 2009 dengue guidelines.

**Results:**

A total of 590 adult dengue patients were included in the study with the mean age 34.3 ± 12.6 (SD) years, with clear male predominance (73.7%). Common symptoms included fever (92.7%), headache (82.7%), myalgia (76.9%), arthralgia (68.0%), nausea (59.8%), anorexia (44.7%), vomiting (42.5%), and diarrhea (18.3%). Warning signs were present in 31.5% of patients, most frequently abdominal pain (41.2%), lethargy (18.1%), restlessness (17.3%), fluid accumulation (ascites 16.9%, pleural effusion 9.0%), and respiratory distress (14.6%). Severe dengue was identified in 32.7% of cases, characterized by plasma leakage (16.4%), bleeding manifestations (melena 8.5%, hemoptysis 5.3%), neurological symptoms (confusion 5.3%, convulsions 1.2%), and shock (6.8%). Two deaths were recorded.

**Conclusion:**

This study found a predominance of young adult males, with nearly one-third developing severe dengue during the 2025 outbreak in Bangladesh.

## Author summary

Dengue fever is a major public health burden, particularly in tropical and subtropical countries, where millions of infections and thousands of deaths occur annually. Bangladesh has experienced recurrent dengue outbreaks over the past two decades, with increasing numbers of cases, expanding geographic distribution, and rising disease severity in recent years. These outbreaks have imposed a substantial burden on patients and the health system of Bangladesh. Additionally, the clinical and epidemiological characteristics of dengue have been evolving over time, with variations in disease presentation across different outbreaks. Understanding these changing patterns is essential for improving patient management and strengthening outbreak preparedness. In this multicenter observational study, 590 hospital admitted dengue patients during the 2025 outbreak were evaluated for clinical and epidemiological features. Most patients were young adults, and males constituted nearly three-quarters of the study population. Fever, headache, myalgia, arthralgia, nausea, and gastrointestinal symptoms were common clinical manifestations. Approximately one-third of patients presented with warning signs and one-third were classified as severe dengue. Despite of higher proportion of warning signs and severe form of disease, most patients recovered following treatment during discharge. Our findings provide important insights into the clinical and epidemiological profile of the 2025 outbreak and highlight the need for strengthened surveillance, early recognition of severe disease, and improved healthcare preparedness to reduce dengue-related complications and mortality in Bangladesh.

## Introduction

Dengue fever remains a major global health threat, endemic in nearly 170 countries and primarily affecting tropical and subtropical regions worldwide [1]. Recently, an alarming surge in dengue infections has been observed, with 2024 recording the highest global incidence to date - approximately 14.6 million cases and 12000 associated deaths [1]. Bangladesh has been one of the most one of the most severely affected countries, consistently reporting higher dengue fatality rates in 2020, 2021, and notably in 2023 compared to other major dengue-endemic nations [2]. In 2023 alone, 321000 dengue cases and 1705 deaths were reported in Bangladesh, representing a 1.3-fold increase in incidence and two-fold rise in deaths compared to the total incidences and mortality of past 23 years [3,4]. This surge has persisted into 2025 [5,6]. According to the Directorate General of Health Services (DGHS) Dengue Dynamic Dashboard, 101211 cases and 575 deaths were officially reported in 2024; by November 2, 2025, 71675 cases and 283 deaths had been reported [7].

Dengue fever is a viral disease caused by the dengue virus (DENV) and transmitted through Aedes mosquitoes, classically presents as acute febrile illness with typical symptoms including headache, retro-orbital pain, myalgia, arthralgia, rash, and mild hemorrhagic events [8]. Hence, among 390 million annual dengue cases, only 96 million manifest clinically [9]. So, a large proportion of subclinical or asymptomatic dengue infections remain unnoticed, especially in resource limiting settings like Bangladesh. Additionally, the epidemiology of dengue in Bangladesh has been undergoing through profound and multifaceted paradigm shift [3,10].

Previously, dengue outbreaks in Bangladesh were largely confined to urban areas, with DENV-2 as the predominant serotype [11]. From 2019 onwards, DENV-3 has emerged as the dominant serotype along with an increase of incidences and severity and spread of cases from urban Dhaka to previously less-affected rural [11–14]. The clinical spectrum has also showed a changing pattern with the earlier outbreaks marked with classical dengue fever with mild hemorrhagic manifestations [10]. Recent outbreaks were marked with a higher frequency of gastrointestinal symptoms and prominent bleeding manifestations along with an increasing incidence of disease severity [10,15–17]. In 2023, 67% of hospitalized patients died within first day of hospitalization [3].

Given the evolving clinical presentation and increasing dengue burden in Bangladesh, updated hospital-based data are needed to guide clinical recognition, patient triage, and outbreak preparedness. This study, therefore, aimed to comprehensively evaluate the clinical and epidemiological characteristics of the 2025 dengue outbreak in Bangladesh.

## Methods

### Ethics statement

The study was approved by the Ethical Review Committee of Public Health Foundation **(PHFBD-ERC-NFP-E-29/2025)**. All the authors declare that no human subjects were harmed and that the procedures followed were in accordance with the ethical standards and regulations established by the Helsinki Declaration of the World Medical Association. Formal permission was taken from respective authority of all study hospitals prior data collection. Written informed consent was obtained from all participants.

### Study design and setting

This observational study was conducted from June to October 2025 across four major tertiary care hospitals representing three administrative divisions of Bangladesh. These included Dhaka Medical College Hospital (DMCH) and Delta Medical College Hospital (DMCHL) in Dhaka division, Mymensingh Medical College Hospital (MMCH) in Mymensingh division, and Chittagong Medical College Hospital (CMCH) in Chittagong division. This multi-center design was adopted to comprehensively capture patients from three urban referral centers during the peak transmission season of the outbreak.

### Study population and sample size

Hospitalized adult patients who were residents of Bangladesh with laboratory-confirmed dengue infection admitted to the participating hospitals during the study period were eligible for enrollment. According to the 2009 World Health Organization (WHO) dengue guidelines, dengue diagnosis was confirmed based on either a positive NS1 antigen test or dengue-specific IgM antibody test. Patients with co-infections (e.g., malaria, typhoid), chronic liver or kidney disease unrelated to dengue were excluded from this study

The minimum required sample size was estimated as 384 using a single population proportion formula with a 95% confidence level, 5% margin of error, and an assumed prevalence of 50%. After applying a design effect of 1.5 for multicenter recruitment, the adjusted minimum sample size was 576. Consecutive eligible patients were enrolled throughout the predefined study period and finally 590 patients were enrolled.

### Development of research tool

For data collection, a semi-structured case record from (CRF) was constructed. The CRF contained all required information including demographic information, clinical and laboratory parameter, in-hospital outcome. The CRF was pretested among 25 dengue patients and adjusted accordingly.

### Face-to-face interview and history taking

Patients were evaluated on the day of admission. Data were collected through face-to-face interviews conducted by trained research assistants. History was taken regarding demographics, symptoms, medical history, comorbidities, and recent travel history. In cases where patients were unable to respond due to severity of illness, altered consciousness, or other incapacitating conditions, reliable attendants or caregivers accompanying the patients were interviewed to provide the necessary information.

Duration of illness was defined as the interval between the onset of fever and hospital presentation. This information was obtained from patient history at the time of admission and recorded in days.

### General and systemic physical examination

A thorough physical examination was performed on each patient by experienced clinicians, following standardized clinical protocols. General examination included assessment of the patient’s overall appearance, vital signs (temperature, pulse, blood pressure, respiratory rate), and nutritional status. Physical characteristics such as body habitus, posture, and signs of distress were noted. The systemic examination encompassed detailed evaluation of major organ systems: cardiovascular, respiratory, abdominal, neurological, and integumentary. Findings were recorded with additional notes if needed.

### Laboratory investigations

Blood samples were collected for laboratory test. Complete blood count and liver function tests were conducted. An automated hematology analyzer, Siemens ADVIA 2120 (Munich, Germany: Siemens) was used is assessment of complete blood count. Semiautomated biochemistry analyzer (Humalyzer 3000, Human Diagnostics, Germany) with commercially available reagent kits was used in assessment of liver function tests.

Face-to-face interview, physical examination and laboratory investigation were performed on the day of admission. In-hospital outcome of the patients were also recorded at discharge or referral.

### Disease severity classification

Patients were classified according to the revised WHO 2009 dengue case classification.[18] Though this classification, confirmed dengue cases are classified intro three groups- dengue cases without warning signs, dengue cases with warning signs and severe dengue cases. In this study patients without warning signs and patients with warning signs were considered as non-severe dengue.

Warning signs include- abdominal pain or tenderness, persistent vomiting, clinical fluid accumulation, mucosal bleeding, lethargy, restlessness, liver enlargement >2 cm, or an increase in hematocrit (>20%) concurrent with a rapid decrease in platelet count. Severe dengue is characterized by severe plasma leakage, severe bleeding, severe organ impairment, or significant metabolic or electrolyte abnormalities.

### Clinical assessment and outcome ascertainment

Clinical symptoms, physical examination findings, and laboratory parameters were recorded at the time of hospital admission. As this was a hospital-based observational study, outcome assessment was limited to in-hospital disposition. However, we will collect most of the data at day of admission and no more follow-up were done. Outcome data were collected from hospital records. Outcomes were categorized as discharge with advice, discharge on risk bond, absconded, referral to a high-dependency unit, referral to an intensive care unit, or death. Mortality or death refers to in-hospital deaths occurring during the hospitalization period.

### Body mass index

The Body mass index (BMI) of the study participants was categorized using the Asian-specific BMI cutoff values recommended by the World Health Organization (WHO). According to these criteria, individuals with a BMI of <18.5 kg/m² were classified as underweight, those with a BMI of 18.5–22.9 kg/m² as having normal weight, 23.0–24.9 kg/m² as overweight, and ≥25.0 kg/m² as obese [19].

### Statistical analysis

The data were analyzed via IBM SPSS Statistics (IBM Corp., Armonk, NY, USA; version 26.0). Descriptive statistics were used to summarize the demographic and clinical characteristics of the study population. Continuous data were assessed for normality through Shapiro-Wilk test while data with normal distribution were reported as the means ± standard deviations (SDs) and data with non-parametric distribution were presented as median (minimum-maximum). Categorical data were presented as frequencies and percentages. Study patients were categorized into non-severe dengue and severe dengue groups according to the revised WHO 2009 dengue classification. Bivariate analyses were performed to compare demographic, clinical, comorbidity-related, and laboratory variables between the two groups. Categorical variables were compared using the chi-square test or Fisher’s exact test, as appropriate. Continuous variables were compared using the independent samples t-test or Mann–Whitney U test depending on data distribution. Univariable logistic regression was performed to estimate crude odds ratios for factors associated with severe dengue. Variables with clinical relevance and/or p < 0.20 in univariable analysis were considered for inclusion in the multivariable logistic regression model. Clinical features used to define warning signs or severe dengue were not included as predictors in the regression model to avoid circular interpretation. Adjusted odds ratios with 95% confidence intervals were reported. A heat map was generated to visualize the prevalence of selected clinical features across dengue severity categories. A p-value <0.05 was considered statistically significant.

## Result

A total of 590 hospitalized adult patients with laboratory-confirmed dengue infection were enrolled from the four participating tertiary care hospitals during the study period. The mean age of the studied dengue patients was 34.3 ± 12.6 (SD) years and nearly half were young adults (18–30 years). Male patients are predominant with a percentage of 73.7% and most resided in urban areas. The average monthly income was 32099 ± 14992.9 (SD) Bangladeshi Taka. In terms of nutritional status, 52% had normal BMI, while 22.4% were overweight and 19.6% obese. Common comorbidities included diabetes mellitus (15.8%) and hypertension (13.6%). Approximately one-third (31.2%) reported recent travel within 30 days (Table 1**).** The most common symptoms among the study patients were fever, headache, myalgia, arthralgia, nausea, anorexia,acute vomiting and diarrhea. Common warning signs included abdominal pain, lethargy, restlessness, ascites, abdominal distension, respiratory distress, pleural effusion and hepatomegaly. Severe manifestations included plasma leakage, shock, bleeding manifestations, neurological symptoms, jaundice, edema, and cold clammy extremities.

**Table 1:** Baseline characteristics of the study participants diagnosed with dengue (N = 590).

Characteristics of the patients	n (%)
**Age group (years) (n = 582)**	
18-30	289 (49.7)
31-40	142 (24.4)
41-50	84 (14.4)
51-60	48 (8.2)
61-70	12 (2.2)
>70	6 (1.0)
Mean±SD*	34.3 ± 12.6
**Gender (n = 589)**	
Male	434 (73.7)
Female	155 (26.3)
**Residence (n = 584)**	
Urban	446 (75.6)
Rural	138 (23.4)
**Monthly family income (n = 466)**	
Mean±SD (Bangladesh Taka)*	32098.7 ± 14992.9
**BMI category (n = 550)**	
Underweight	33 (6.0)
Normal	286 (52.0)
Overweight	123 (22.4)
Obese	108 (19.6)
Mean±SD (kg/m^2^)*	22.7 ± 3.4
**Comorbidities**	
Diabetes Mellitus	93 (15.8)
Hypertension	80 (13.6)
Peptic Ulcer Disease	42 (7.1)
Hypothyroidism	7 (1.2)
Bronchial Asthma	5 (0.8)
Chronic Kidney Disease	4 (0.7)
**Treating facility**	
Dhaka Medical College Hospital	182 (30.8)
Delta Medical College Hospital	97 (16.4)
Mymensingh Medical College Hospital	191 (32.4)
Chittagong Medical College Hospital	120 (20.3)
**History of travelling in last 30 days**	177 (31.2)

BMI: body mass index.

Percentages were calculated as valid percentages excluding missing data for each variable.

*Data are presented as mean ± standard deviation.

Laboratory results showed a mean hematocrit of 38.8 ± 6.1 (SD)%, median hemoglobin of 12.8 g/dl, median white blood cell count of 5435/µL, and median platelet count of 80000/µL. Median of alanine aminotransferase (ALT) and aspartate aminotransferase (AST) were 66 U/L and 88.5 U/L.

Regarding severity classification, 67.3% of patients had non-severe dengue, with 35.8% without warning signs and 31.5% with warning signs. About 32.7% were classified as severe dengue (Table 2).

**Table 2:** Clinical presentations and dengue severity of the study participants on the day of admission (N = 590).

Clinical presentations	n (%)
**Clinical features**	
Fever	547 (92.7)
Duration from onset of fever (days) at the time of admission(n = 583)	
≤ 3	110 (18.9)
4-6	262 (44.9)
≥ 7	211 (36.2)
Mean±SD	6.1 ± 3.2
Headache	488 (82.7)
Myalgia	453 (76.9)
Arthralgia	401 (68)
Nausea	353 (59.8)
Anorexia	264 (44.7)
Acute vomiting	251 (42.5)
Cough	198 (33.6)
Redness of eyeat	131 (22.2)
Chest pain	120 (20.3)
Diarrhea	108 (18.3)
Palpitation	93 (15.8)
Dizziness	80 (13.6)
Rash	68 (11.7)
**Warning signs**	
Abdominal pain	243 (41.2)
Lethargy	107 (18.1)
Restlessness	102 (17.3)
Ascites	100 (16.9)
Abdominal distension	91 (15.4)
Respiratory distress	86 (14.6)
Gum bleeding	77 (13.1)
Pleural effusion	53 (9.0)
Persistent vomiting	36 (6.1)
Epistaxis	25 (4.2)
Hepatomegaly	25 (4.2)
**Signs of sever dengue**	
Features of plasma leakage	97 (16.4)
Pale and cold clammy hands and feet	67 (11.4)
Melena	50 (8.5)
Shock	40 (6.8)
Hemoptysis	31 (5.3)
Confusion	31 (5.3)
Excessive menstrual bleeding	22 (3.7)
Loss of consciousness	22 (3.7)
Jaundice	21 (3.6)
Hematemesis	19 (3.2)
Hematuria	7 (1.2)
Convulsion	7 (1.2)
Edema	16 (2.7)
**Laboratory findings**	
Hematocrit (%)*	38.8 ± 6.1
Hemoglobin (g/dl)**	12.8 (7.1-17.8)
White blood cell (cells/µL)**	5435 (1700-145000)
Platelet (cells/µL)**	80000 (2000-465000)
Alanine Aminotransferase (U/L)**	66 (12-702)
Aspartate Aminotransferase (U/L)**	88.5 (15-906)
**Severity of dengue**	
Non-severe dengue	397 (67.3)
Dengue without warning signs	211 (35.8)
Dengue with warning signs	186 (31.5)
Severe dengue	193 (32.7)

Data were presented as mean ± standard deviation* and median (minimum-maximum)**

The in-hospital outcomes of the 447 study participants were recorded while 93.5% were discharged with advice. About 2.2% were discharged on risk bond and 1% were absconded. About 6 patients were referred to higher dependency units (HDU), 2 were referred to intensive care unit (ICU) while only 2 patients died (Table 3).

**Table 3:** In-hospital outcome of the study participants (N = 447).

In-hospital outcome	n (%)
Discharge with advice	418 (93.5)
Discharge on risk bond	13 (2.2)
Absconded	6 (1)
Referred to HUD	6 (1)
Referred to ICU	2 (.3)
Death	2 (.3)

Percentages were calculated as valid percentages excluding missing data

Additional comparative analysis was performed to identify factors associated with severe dengue. The distribution of age group, sex, residence, and BMI category did not differ significantly between patients with non-severe and severe dengue. However, duration of illness before hospital admission was significantly associated with disease severity. A higher proportion of patients with severe dengue presented after seven or more days of illness compared with those with non-severe dengue. The median duration of illness was also significantly longer among severe dengue patients than non-severe dengue patients. History of recent travel within the preceding 30 days differed significantly between the two groups. Among comorbidities, peptic ulcer disease was significantly more frequent among patients with severe dengue, while hypertension and diabetes mellitus did not show statistically significant differences between the severity groups. Laboratory comparison showed that severe dengue patients had significantly lower white blood cell and platelet counts than non-severe dengue patients. Hematocrit, hemoglobin, ALT, and AST levels did not differ significantly between the two groups (Table 4).

**Table 4:** Factors associated with severe dengue among hospitalized dengue patients.

	Non-severe dengue n (%)	Severe dengue n (%)	p-value
**Demographic variables**			
Age group			
18–30	204 (52.3)	85 (44.3)	0.385**
31–40	88 (22.6)	54 (28.1)	
41–50	52 (13.3)	32 (16.7)	
51–60	33 (8.5)	15 (7.8)	
61–70	8 (2.1)	5 (2.6)	
>70	5 (1.3)	1 (0.5)	
Mean±SD	33.8 ± 12.8	35.1 ± 12.2	0.643^∆^
Sex			
Male	295 (74.5)	139 (72.0)	0.589*
Female	101 (25.5)	54 (28.0)	
Residence			
Urban	306 (78.1)	140 (72.9)	0.204*
Rural	86 (21.9)	52 (27.1)	
BMI category			
Underweight	20 (5.5)	13 (6.9)	0.772*
Normal	189 (52.4)	97 (51.3)	
Overweight	84 (23.3)	39 (20.6)	
Obese	68 (18.8)	40 (21.2)	
Duration of illness during hospital admission			
≤3 days	89 (22.7)	21 (11.0)	0.002*
4–6 days	172 (43.9)	90 (47.1)	
≥7 days	131 (33.4)	80 (41.9)	
Median (IQR)	5.0 (4.0-7.0)	6.0 (5.0-7.0)	.004^∆∆^
**History of recent travel**	129 (34.1)	48 (25.3)	0.040*
**Presence of comorbidity**			
Peptic ulcer disease	14 (3.5)	28 (14.5)	<0.001*
Hypertension	48 (12.1)	32 (16.6)	0.172*
Diabetes mellitus	56 (14.1)	37 (19.2)	0.143*
**Laboratory variables**			
Hematocrit (%)	38.5 (35.8-42.0)	38.5 (34.0-43.3)	0.496^∆∆^.
Hemoglobin (g/dL)	12.8 (11.8-14.0)	12.8 (11.2-14.2)	0.665^∆∆^.
White blood cell (cell/µL)	6000 (4293–9000)	4600 (3200–6950)	<0.001^∆∆^.
Platelet (cell/µL)	95000 (45000–150000)	60000 (32000–110000)	<0.001^∆∆^.
ALT (U/L)	65.0 (45.0-134.0)	68.0 (40.2-125.0)	0.653^∆∆^.
AST (U/L)	96.0 (65.5-140.0)	86.0 (54.5–123.5)	0.304^∆∆^.

Data are presented as n (%) unless otherwise stated. Percentages were calculated using valid data after excluding missing values for each variable. Continuous variables are presented as mean ± standard deviation or median (interquartile range), as appropriate. Categorical variables were compared using the chi-square test* and Fisher’s exact test**, and continuous variables were compared using the independent samples t-test^∆^ and Mann–Whitney U test^∆∆^.

BMI: body mass index; ALT: alanine aminotransferase; AST: aspartate aminotransferase; IQR: interquartile range.

Univariable logistic regression showed that recent travel history and peptic ulcer disease, and study hospital were significantly associated with severe dengue. In the multivariable logistic regression model, peptic ulcer disease remained independently associated with severe dengue after adjustment for age, sex, residence, duration from fever onset, recent travel history, hypertension and diabetes mellitus (Table 5).

**Table 5:** Univariate and multivariate logistic regression for factors associated with severe dengue.

Variables	Univariate		Multivariate	
	**OR (95% CI)**	**p-value**	**OR (95% CI)**	**p-value**
Age (year)	1.01 (0.99–1.02)	0.259	1.00 (0.98–1.02)	0.859
Male sex	0.88 (0.60–1.30)	0.522	0.97 (0.60–1.57)	0.900
Rural residence	1.32 (0.89–1.97)	0.170	1.20 (0.73–1.98)	0.479
Duration of illness on the day of admission (day)	1.04 (0.99–1.10)	0.103	1.02 (0.96–1.09)	0.476
History of recent travel	0.65 (0.44–0.96)	0.032	1.38 (0.71–2.67)	0.344
Peptic ulcer disease	4.64 (2.38–9.05)	<0.001	2.69 (1.23–5.89)	0.013
Hypertension	1.45 (0.89–2.35)	0.137	1.72 (0.87–3.41)	0.121
Diabetes mellitus	1.44 (0.92–2.28)	0.114	1.60 (0.84–3.05)	0.153

OR: odds ratio; AOR: adjusted odds ratio; CI: confidence interval; Severe dengue was coded as the outcome variable, with non-severe dengue as the reference category. Female sex, urban residence, no recent travel, absence of comorbidity were used as reference categories. The multivariable model included age, sex, residence, duration from fever onset, recent travel history, peptic ulcer disease, hypertension and diabetes mellitus, and study hospital. Clinical features used to define warning signs or severe dengue were not included as predictors to avoid circular interpretation.

**Fig 1:**
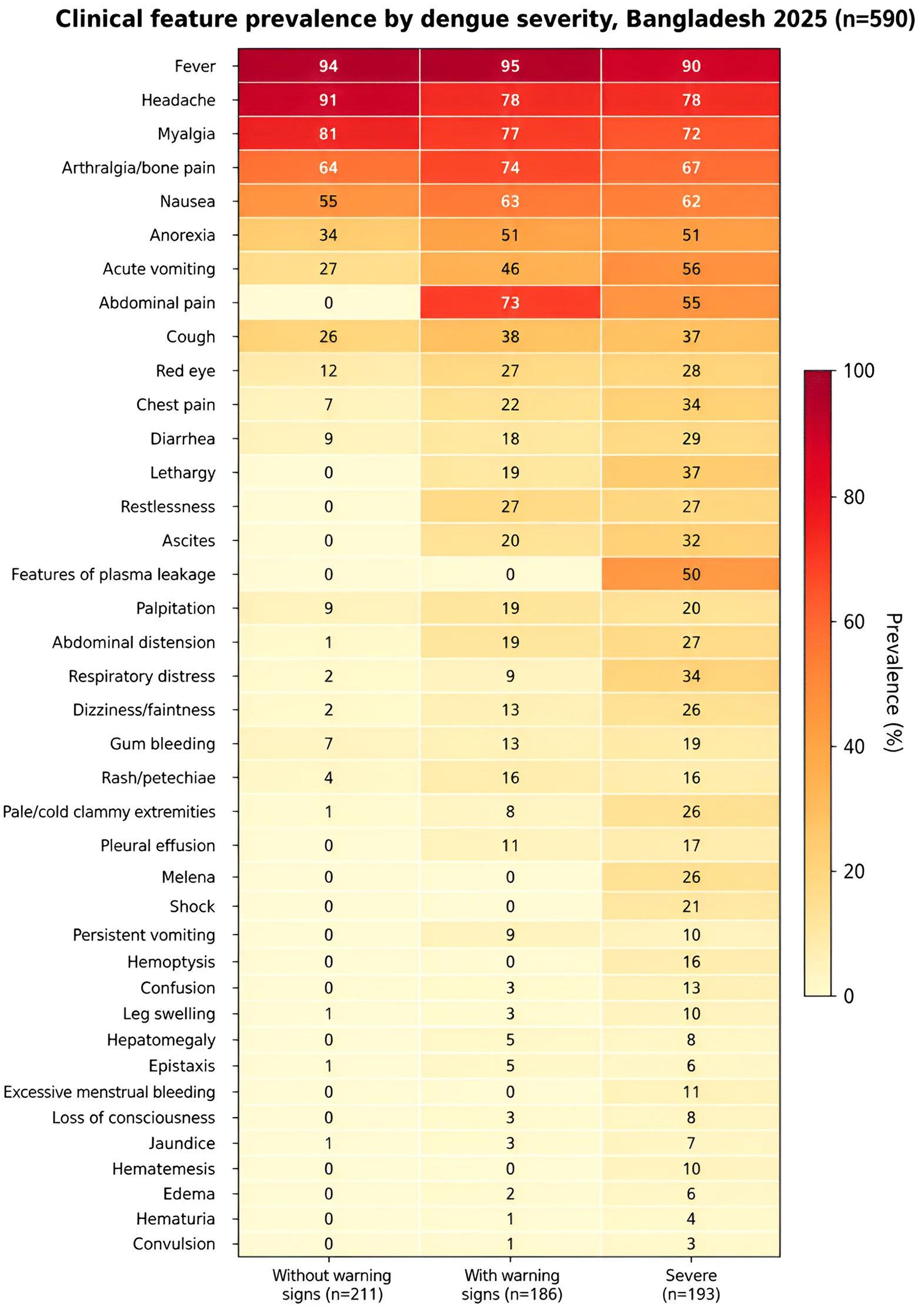
Heat map of clinical feature prevalence by dengue severity among hospitalized dengue patients during the 2025 outbreak in Bangladesh.

The distribution of clinical features across dengue severity categories is shown in Fig 1. Classical dengue symptoms, including fever, headache, myalgia, arthralgia, and nausea, were common across all severity groups. Gastrointestinal symptoms, particularly acute vomiting and abdominal pain, were more frequent among patients with warning signs and severe dengue. Features suggestive of complicated disease, including plasma leakage, respiratory distress, ascites, melena, shock, hemoptysis, confusion, and hematemesis, were predominantly observed among patients with severe dengue.

## Discussion

Dengue fever continues to exhibit changes in epidemiological and clinical trends over the decades [10,11]. The 2025 dengue outbreak in Bangladesh is also being marked with increasing cases and mortality [20,21]. This observational study described the clinical spectrum of confirmed dengue cases from four tertiary care hospitals from three divisions of the country.

In this study at total of 590 patients with confirmed dengue fever were studied. The average age of the patients was 34 years, with the majority being young adults. Clear male predominance was observed with almost three-fourth of the study patients as male. This pattern characterized by predominance of male young adults was observed in prior outbreaks in Bangladesh and other dengue-endemic countries [22,23]. The predominance of young adults is likely due to their increased mobility and social activity, which elevate their exposure to Aedes mosquito bites. Three-fourth of the study patients hailed from urban area which points out to expand of infection from urban to rural area of the country observed since 2019 [13,14]. Approximately 31.2% of patients reported recent travel within the preceding 30 days, indicating that population mobility may be an important contextual factor to consider in dengue outbreak surveillance.

Fever, headache, myalgia, and arthralgia were the most reported symptoms among the studied patients which mirrors the typical dengue presentations globally [24]. Alongside the typical symptoms, higher frequency of gastrointestinal symptoms was observed including acute vomiting and diarrhea. Among warning signs- abdominal pain and lethargy were the most common. Among signs of severe dengue- shock, confusion, convulsion, pale and cold clammy hands, edema, forms of severe bleeding, features of plasma leakage were reported. These presentation points toward a shift in clinical spectrum characterized by pronounced gastrointestinal involvement and increased severity -reported in 2023 and 2024 outbreaks in Bangladesh [3,22,23,25,26].

Laboratory findings was consistent with the expected hematological involvement of dengue infection. Thrombocytopenia was prominent, while the hematocrit and hemoglobin levels suggested possible hemoconcentration in some patients, supporting the clinical importance of monitoring for plasma leakage. The white blood cell count also reflected the leukopenic tendency commonly observed in dengue. A high proportion of hospitalized patients presented with warning signs or severe dengue, with each category accounting for approximately one-third of cases. This pattern appears higher compared to earlier outbreaks, with severe dengue documented in 9% of patients during the 2024 outbreak and 20% in the 2023 outbreak. As this study was conducted in tertiary care hospitals, the severity profile observed should be interpreted in the appropriate context. While tertiary care settings typically attract more complicated cases, it is important to note that in Bangladesh, a substantial proportion of patients present directly to tertiary hospitals without formal referral from lower-level facilities [27]. This pattern of direct self-presentation suggests that our sample likely includes a broader clinical spectrum of dengue patients, including those with mild to moderate illness, rather than exclusively representing severe cases filtered upward through a structured referral pathway. Nevertheless, we acknowledge that patients with no or mild symptoms may still preferentially seek care at primary healthcare facilities or community-level centres, and the observed proportion of warning signs and severe dengue may be somewhat higher than that of the overall dengue-affected population in Bangladesh. In this study, obesity, diabetes mellitus, and hypertension were observed among a notable proportion of the study patients with dengue fever. –These are common factors exacerbating disease severity by compromising immune response and organ function. Presence of these comorbidities also may play role in increasing severity and complicating clinical courses over recent years along with serotype evolution and host immune factors. Delay in diagnosis and intervention might be another reason behind this increasing severity.

In this study, about 93.5% of patients were successfully discharged following clinical management, with only 2 patients recorded as in-hospital mortalities and 2 patients each referred to the intensive care unit (ICU) and high dependency unit (HDU). The dengue mortality rate in this study is relatively low compared to previous years in Bangladesh, where mortality varied notably: approximately 0.9% in 2021, 1.2% in 2022, and surged up to 2.8% in the severe 2023 outbreak, followed by a reduced rate around 0.7% in 2024 [28,29]. However, it is important to note that the year 2025 is not yet over, and many patients who absconded or were discharged on risk bonds have not been fully tracked, making it likely that the true mortality is underestimated. This situation indicates gaps in patient follow-up, underreporting, and challenges in comprehensive surveillance, which obscure the real burden of dengue mortality in Bangladesh.

From a public health perspective, the higher proportion of hospitalized patients presenting with warning signs or severe dengue highlights the need for strengthened outbreak preparedness in Bangladesh. Also, fewer number of mortality and ICU admission points towards the importance of early recognition of warning signs at primary and secondary healthcare levels. Ensuring timely referral to appropriate facilities and adequate preparation of tertiary hospitals are essential during peak transmission periods. Integration of hospital-based clinical data with routine surveillance may also help health authorities identify changes in disease severity and allocate resources more effectively during future outbreaks.

This study was not beyond limitations. First, participants were recruited from four tertiary care hospitals; therefore, the findings may not represent the full spectrum of dengue cases in the community. Patients with no or mild symptoms may have not seek care or have been managed at primary healthcare facilities, outpatient departments, or community-level centers not requiring admission to tertiary hospitals. As a result, the proportion of warning signs and severe dengue observed in this study may be somewhat higher than that of the overall dengue-affected population in Bangladesh during the 2025 outbreak, though this effect may be partially mitigated by the high rate of self-referral to tertiary hospitals documented in the Bangladesh healthcare context. Second, dengue virus serotyping and genotyping could not be performed because of limited funding and laboratory resource constraints; therefore, associations between circulating viral serotypes or genotypes and disease severity could not be assessed. Third, some clinical features, including retro-orbital pain, were not systematically captured as separate variables in the case record form and therefore could not be analyzed. Finally, outcome assessment was limited to in-hospital disposition, and post-discharge outcomes were not evaluated. Expanding the study to include more hospitals and community-based surveillance would provide a more representative picture of dengue epidemiology and severity across different regions. Incorporating virological characterization would help clarify the relationship between circulating dengue virus serotypes, clinical presentation, and disease severity.

## Conclusion

This study provides insights into the clinical and epidemiological profile of dengue patients during the 2025 outbreak in Bangladesh. A predominance of young adult males, a high prevalence of patients with warning signs and severe dengue were reported in this outbreak. However, as the study was conducted in tertiary care hospitals, the observed severity profile should be interpreted as reflecting hospitalized dengue cases rather than all dengue infections in the community. These findings highlight the need for early recognition of warning signs, timely referral, strengthened hospital preparedness, and improved surveillance to reduce dengue-related complications and mortality in Bangladesh. Understanding this paradigm shift is crucial for adapting surveillance systems, refining clinical protocols, and implementing targeted vector control strategies to mitigate the ongoing crisis in Bangladesh and other densely populated, resource-limited settings

## Supporting information

S1 File De-identified patient-level dataset and codebook underlying the analyses reported in this study. The dataset includes demographic characteristics, comorbidities, clinical manifestations, laboratory findings, dengue severity classification according to the 2009 World Health Organization (WHO) dengue guidelines, and in-hospital outcomes of hospitalized adults with laboratory-confirmed dengue infection during the 2025 dengue outbreak in Bangladesh. The accompanying codebook provides variable names, definitions, and coding used in the dataset.
(XLSX)
