# Supplementary material for: Clinical and epidemiological characteristics of the 2025 dengue outbreak in Bangladesh
Source: PLoS Negl Trop Dis. 2026 Aug 26;20(8):e0014661. doi: 10.1371/journal.pntd.0014661 (PMC13577480; doi:10.1371/journal.pntd.0014661)
Supplement: Supplementary file 1 [file pntd.0014661.s004.pdf]

July 05, 2026

To,
The Editor-in-Chief
PLOS Neglected Tropical Diseases

Re: Submission of the revised manuscript '*Clinical and Epidemiological Characteristics of the 2025 Dengue Outbreak in Bangladesh*' [PNTD-D-25-02195]

Dear Editorial Team,

Thank you for your email outlining the technical issues identified during the initial assessment of our manuscript. We appreciate the careful review of our submission.

We have addressed all of the requested modifications. Thank you for your time and consideration. We look forward to your response.

Yours sincerely,

Tamanna Tabassum
Research Investigator, Pi Research & Development Center, Dhaka, Bangladesh
dr.tabassum1991@gmail.com

Response to technical check comments

We sincerely thank the Editorial Office for carefully reviewing our submission. We have addressed each of the technical issues as outlined below.

Comment 1

We have amended your Competing Interest statement to comply with journal style. Please check the statement and let us know if anything is incorrect.

Response: *Thank you. We have carefully reviewed the amended Competing Interest statement and confirm that it is accurate.*

Comment 2

Please upload all main figures as separate Figure files in .tif or .eps format.

Response: *Thank you. All main figures have now been uploaded separately in the required file format.*

Comment 3

Please upload a copy of Figure 1 referred to in the manuscript (line 282), or remove all references to it if it is no longer included.

Response: *Thank you. Figure 1 has now been uploaded as a separate figure file and is correctly cited in the revised manuscript.*

Comment 4

Please add a legend for the Supporting Information file after the References section.

Response: *Thank you. A legend describing the Supporting Information file has been added immediately after the References section in the revised manuscript.*

Comment 5

Please revise the Data Availability Statement so that data requests are directed to a non-author institutional contact and provide the relevant contact information.

Response: *Thank you. The Data Availability Statement has been revised.*

Comment 6

Please ensure that the Supporting Information dataset contains no identifying information and upload a fully anonymized version.

Response: *Thank you. The Supporting Information dataset has been thoroughly reviewed and fully anonymized. All direct and indirect personal identifiers have been removed in accordance with participant confidentiality requirements, applicable ethical standards, and the journal's Data Policy. The anonymized dataset has been uploaded as the supplementary file 1.*
