# Supplementary material for: Clinical and epidemiological characteristics of the 2025 dengue outbreak in Bangladesh
Source: PLoS Negl Trop Dis. 2026 Aug 26;20(8):e0014661. doi: 10.1371/journal.pntd.0014661 (PMC13577480; doi:10.1371/journal.pntd.0014661)
Supplement: Supplementary file 2 [file pntd.0014661.s002.docx]

**Review Report**

The work presented is interesting and helps to describe the history of dengue fever in Bangladesh. The manuscript has some gaps that need to be clarified for a better understanding of the study.

Below are some suggestions and considerations that could improve the quality of the work:

**Abstract**

Information regarding the total number of patients included is not part of the "Methods" section. It should be in the "Results" section.

It is very important to state in the summary at what stage of the disease the patients were evaluated (days of disease progression).

**Keywords**

While possible, the yearWhile possible, the year of the study is not recommended as a primary choice unless the specific time period is crucial for understanding or locating the research. I would recommend removing "2025" from keywords. of the study is not recommended as a primary choice unless the specific time period is crucial for understanding or locating the research. I would recommend removing "2025" from keywords.

"Clinical Spectrum" doesn't seem like an appropriate descriptor. I would recommend "symptoms".

It would also be very important to have a keyword related to the study design. I would recommend "cohort study".

**Introduction**

This sentence is not part of an introduction: "**Understanding this paradigm shift is crucial for adapting surveillance systems, refining clinical protocols, and implementing targeted vector control strategies to mitigate the ongoing crisis in Bangladesh and other densely populated, resource-limited settings**." (lines 111-113). Since no reference was provided, it appears to be a conclusion of the authors and should be in the conclusions section of this study.

**Methods**

This information is part of the results: "**A total of 590 patients diagnosed with confirmed dengue cases and admitted to study hospitals were enrolled in this study**." I recommend including this information in the results.

The time frame for disease progression (in days) is a very important clinical detail. Did the study not consider this data?

**Results**

What was the nationality of these patients? Were they all from Bangladesh?

Were these patients evaluated only once? This needs to be clear.

When there is a reference to a death outcome, at what point in time is it referring? Are these people who died after the initial assessment? During hospitalization? This needs to be clear. I understand that these patients did not have follow-up and were assessed at a single point in time.

Table 2 describes the duration of fever (in days). However, these patients were evaluated in a single consultation, without follow-up. How can they describe the duration of the fever? I suggest removing this data.

**Discussion**

In the manuscript's introduction, the authors mention retro-orbital pain as a common symptom in dengue fever. However, this was not observed in the reported study. Don't you think this is an interesting point to report?

**Conclusions**

It is difficult to conclude what percentage of severity in this sample describes the profile of a dengue outbreak in Bangladesh in 2025, as the study was conducted in hospitals and it is expected that the most severe patients will arrive at this type of health unit. Are there basic health units that treat patients with mild symptoms? This is important to mention.

Another major flaw in this study was the failure to report how many days the participants had been experiencing symptoms when they were treated in these hospitals. Were these patients with more prolonged symptoms? More than 5 days of evolution?
