# Supplementary material for: Clinical and epidemiological characteristics of the 2025 dengue outbreak in Bangladesh
Source: PLoS Negl Trop Dis. 2026 Aug 26;20(8):e0014661. doi: 10.1371/journal.pntd.0014661 (PMC13577480; doi:10.1371/journal.pntd.0014661)
Supplement: Peer review history

# Peer review: Decision Letter 0

**DOI:** 10.1371/journal.pntd.0014661.r001

**Authors:** Nikos Vasilakis, Eric Mossel

5 Mar 2026

PNTD-D-25-02195

Clinical and Epidemiological Characteristics of the 2025 Dengue Outbreak in Bangladesh

PLOS Neglected Tropical Diseases

Dear Dr. Tabassum,

Thank you for submitting your manuscript to PLOS Neglected Tropical Diseases. After careful consideration, we feel that it has merit but does not fully meet PLOS Neglected Tropical Diseases's publication criteria as it currently stands. Therefore, we invite you to submit a revised version of the manuscript that addresses the points raised during the review process.

Please submit your revised manuscript within by May 04 2026 11:59PM. If you will need more time than this to complete your revisions, please reply to this message or contact the journal office at plosntds@plos.org. When you're ready to submit your revision, log on to https://www.editorialmanager.com/pntd/ and select the 'Submissions Needing Revision' folder to locate your manuscript file.

Please include the following items when submitting your revised manuscript:

* A letter that responds to each point raised by the editor and reviewer(s). You should upload this letter as a separate file labeled 'Response to Reviewers'. This file does not need to include responses to any formatting updates and technical items listed in the 'Journal Requirements' section below.

* A marked-up copy of your manuscript that highlights changes made to the original version. You should upload this as a separate file labeled 'Revised Manuscript with Track Changes'.

* An unmarked version of your revised paper without tracked changes. You should upload this as a separate file labeled 'Manuscript'.

If you would like to make changes to your financial disclosure, competing interests statement, or data availability statement, please make these updates within the submission form at the time of resubmission. Guidelines for resubmitting your figure files are available below the reviewer comments at the end of this letter.

We look forward to receiving your revised manuscript.

Kind regards,

Eric Mossel, Ph.D.

Academic Editor

PLOS Neglected Tropical Diseases

Nikos Vasilakis

Section Editor

PLOS Neglected Tropical Diseases

Shaden Kamhawi

co-Editor-in-Chief

PLOS Neglected Tropical Diseases

orcid.org/0000-0003-4304-636XX

Paul Brindley

co-Editor-in-Chief

PLOS Neglected Tropical Diseases

orcid.org/0000-0003-1765-0002

**Journal Requirements:**

1) Please provide an Author Summary. This should appear in your manuscript between the Abstract (if applicable) and the Introduction, and should be 150-200 words long. The aim should be to make your findings accessible to a wide audience that includes both scientists and non-scientists. Sample summaries can be found on our website under Submission Guidelines:

https://journals.plos.org/plosntds/s/submission-guidelines#loc-parts-of-a-submission

2) We do not publish any copyright or trademark symbols that usually accompany proprietary names, eg ©,  ®, or TM  (e.g. next to drug or reagent names). Therefore please remove all instances of trademark/copyright symbols throughout the text, including:

- ® on page: 7.

3) In the online submission form, you indicated that "Patient-level data will be available upon request from the corresponding author.". All PLOS journals now require all data underlying the findings described in their manuscript to be freely available to other researchers, either

1. In a public repository

2. Within the manuscript itself

3. Uploaded as supplementary information.

This policy applies to all data except where public deposition would breach compliance with the protocol approved by your research ethics board. If your data cannot be made publicly available for ethical or legal reasons (e.g., public availability would compromise patient privacy), please explain your reasons by return email and your exemption request will be escalated to the editor for approval. Your exemption request will be handled independently and will not hold up the peer review process, but will need to be resolved should your manuscript be accepted for publication. One of the Editorial team will then be in touch if there are any issues.

**Reviewers' Comments:**

Reviewer's Responses to Questions

**Key Review Criteria Required for Acceptance?**

As you describe the new analyses required for acceptance, please consider the following:

**Methods**

-Are the objectives of the study clearly articulated with a clear testable hypothesis stated?

-Is the study design appropriate to address the stated objectives?

-Is the population clearly described and appropriate for the hypothesis being tested?

-Is the sample size sufficient to ensure adequate power to address the hypothesis being tested?

-Were correct statistical analysis used to support conclusions?

-Are there concerns about ethical or regulatory requirements being met?

Reviewer #1: The methodology is satisfactory. Some considerations were suggested in the attached document.

Reviewer #2: - The objectives are clearly articulated;

- The study design in partial;

- The population is clearly described;

- The sample size is not sufficient for testing the hypothesis;

- The statistical analysis is correct, although it has been used on the above-mentioned insufficient sample;

- There are no ethical or regulatory concerns.

Reviewer #3: Yes, the section methods is clear and well written.

**********

**Results**

-Does the analysis presented match the analysis plan?

-Are the results clearly and completely presented?

-Are the figures (Tables, Images) of sufficient quality for clarity?

Reviewer #1: Some considerations were suggested in the attached document.

Reviewer #2: - The presented analysis only partially match the analysis plan;

- The results are clear and completely presented;

- The figures are clear.

Reviewer #3: The results primarily present a descriptive analysis of the data. However, I believe the authors could make better use of this information, as the analysis is limited to a univariate approach. The study does not include bivariate or multivariate analyses, which represents a missed opportunity to provide a more comprehensive explanation of what occurred in Bangladesh during the dengue outbreak.

**********

**Conclusions**

-Are the conclusions supported by the data presented?

-Are the limitations of analysis clearly described?

-Do the authors discuss how these data can be helpful to advance our understanding of the topic under study?

-Is public health relevance addressed?

Reviewer #1: Some considerations were suggested in the attached document.

Reviewer #2: - The conclusions are supported by the presented data;

- The limitation of analysis are partially described.

- The author only vaguely explain the helpfulness of their data;

- The relevance for public health is not adequately addressed given the small sample size.

Reviewer #3: Yes, this section is correct.

**********

**Editorial and Data Presentation Modifications?**

Use this section for editorial suggestions as well as relatively minor modifications of existing data that would enhance clarity. If the only modifications needed are minor and/or editorial, you may wish to recommend “Minor Revision” or “Accept”.

Reviewer #1: Minor Revision

Reviewer #2: (No Response)

Reviewer #3: (No Response)

**********

**Summary and General Comments**

Use this section to provide overall comments, discuss strengths/weaknesses of the study, novelty, significance, general execution and scholarship. You may also include additional comments for the author, including concerns about dual publication, research ethics, or publication ethics. If requesting major revision, please articulate the new experiments that are needed.

Reviewer #1: The work presented is interesting and helps to describe the history of dengue fever in Bangladesh. The manuscript has some gaps that need to be clarified for a better understanding of the study.

Reviewer #2: This manuscript has some flaws:

- the sample is too small and does not adequately represent the Bangladese population affected by dengue (590 on more than 71000);

- the genotypes that determine the most severe form of the disease have not been clearly established;

- already known data are repeated in the comments and the results

- there are no strong evidences to support the idea that males are more affected by the diseases because of their social life.

The authors themselves admitted some of these observations.

Reviewer #3: I consider that the authors should make better use of all the available data to improve the presentation of their results. Currently, the analysis appears too basic, and the data are not explored in sufficient depth to adequately explain what is happening in Bangladesh. Therefore, I believe the authors need to substantially strengthen the statistical analysis. In addition, it would be important to include a heat map presenting cases or relevant figures derived from the dataset to enhance data visualization and interpretation.

**********

PLOS authors have the option to publish the peer review history of their article (what does this mean?). If published, this will include your full peer review and any attached files.

If you choose “no”, your identity will remain anonymous but your review may still be made public.

**Do you want your identity to be public for this peer review?**  For information about this choice, including consent withdrawal, please see our Privacy Policy.

Reviewer #1: **Yes:** The work presented is interesting and helps to describe the history of dengue fever in Bangladesh and Asia.

Reviewer #2: No

Reviewer #3: No

[NOTE: If reviewer comments were submitted as an attachment file, they will be attached to this email and accessible via the submission site. Please log into your account, locate the manuscript record, and check for the action link "View Attachments". If this link does not appear, there are no attachment files.]

**Figure resubmission:**  While revising your submission, we strongly recommend that you use PLOS’s NAAS tool (https://ngplosjournals.pagemajik.ai/artanalysis) to test your figure files. NAAS can convert your figure files to the TIFF file type and meet basic requirements (such as print size, resolution), or provide you with a report on issues that do not meet our requirements and that NAAS cannot fix.

After uploading your figures to PLOS’s NAAS tool - https://ngplosjournals.pagemajik.ai/artanalysis, NAAS will process the files provided and display the results in the "Uploaded Files" section of the page as the processing is complete. If the uploaded figures meet our requirements (or NAAS is able to fix the files to meet our requirements), the figure will be marked as "fixed" above. If NAAS is unable to fix the files, a red "failed" label will appear above. When NAAS has confirmed that the figure files meet our requirements, please download the file via the download option, and include these NAAS processed figure files when submitting your revised manuscript.

**Reproducibility:**

To enhance the reproducibility of your results, we recommend that authors of applicable studies deposit laboratory protocols in protocols.io, where a protocol can be assigned its own identifier (DOI) such that it can be cited independently in the future. Additionally, PLOS ONE offers an option to publish peer-reviewed clinical study protocols. Read more information on sharing protocols at https://plos.org/protocols?utm_medium=editorial-email&utm_source=authorletters&utm_campaign=protocols

Attachment Submitted filename: Report.docx

# Author comment: Author response to Decision Letter 1

**DOI:** 10.1371/journal.pntd.0014661.r002

5 Jul 2026

Attachment Submitted filename: 5000191. Revisionnote_technicalcheck_v11-tt.pdf

# Peer review: Decision Letter 1

**DOI:** 10.1371/journal.pntd.0014661.r003

**Authors:** Eric Mossel, Sujatha Sunil

10 Aug 2026

Dear Dr Tabassum,

We are pleased to inform you that your manuscript 'Clinical and Epidemiological Characteristics of the 2025 Dengue Outbreak in Bangladesh' has been provisionally accepted for publication in PLOS Neglected Tropical Diseases.

Before your manuscript can be formally accepted you will need to complete some formatting changes, which you will receive in a follow up email. A member of our team will be in touch with a set of requests.

Please note that your manuscript will not be scheduled for publication until you have made the required changes, so a swift response is appreciated.

IMPORTANT: The editorial review process is now complete. PLOS will only permit corrections to spelling, formatting or significant scientific errors from this point onwards. Requests for major changes, or any which affect the scientific understanding of your work, will cause delays to the publication date of your manuscript.

Should you, your institution's press office or the journal office choose to press release your paper, you will automatically be opted out of early publication. We ask that you notify us now if you or your institution is planning to press release the article. All press must be co-ordinated with PLOS.

Thank you again for supporting Open Access publishing; we are looking forward to publishing your work in PLOS Neglected Tropical Diseases.

Best regards,

Eric Mossel, Ph.D.

Academic Editor

PLOS Neglected Tropical Diseases

Sujatha Sunil

Section Editor

PLOS Neglected Tropical Diseases

Shaden Kamhawi

co-Editor-in-Chief

PLOS Neglected Tropical Diseases

orcid.org/0000-0003-4304-636XX

Paul Brindley

co-Editor-in-Chief

PLOS Neglected Tropical Diseases

orcid.org/0000-0003-1765-0002

***********************************************************

Reviewer's Responses to Questions

**Key Review Criteria Required for Acceptance?**

As you describe the new analyses required for acceptance, please consider the following:

**Methods**

-Are the objectives of the study clearly articulated with a clear testable hypothesis stated?

-Is the study design appropriate to address the stated objectives?

-Is the population clearly described and appropriate for the hypothesis being tested?

-Is the sample size sufficient to ensure adequate power to address the hypothesis being tested?

-Were correct statistical analysis used to support conclusions?

-Are there concerns about ethical or regulatory requirements being met?

Reviewer #1: After careful evaluation, I do not consider any additional analyses or methodological revisions necessary for acceptance. The study objectives are clearly articulated, and the research question is adequately defined. The study design is appropriate to address the stated objectives, the study population is clearly described and suitable for the research question, and the sample size is adequate to support the analyses performed. The statistical methods are appropriate and correctly applied, and I identified no significant concerns regarding ethical or regulatory requirements. Overall, the methodology is sound and adequately supports the conclusions of the study, and no further methodological revisions are required for acceptance.

**********

**Results**

-Does the analysis presented match the analysis plan?

-Are the results clearly and completely presented?

-Are the figures (Tables, Images) of sufficient quality for clarity?

Reviewer #1: The analyses presented are consistent with the study objectives and the reported analytical approach. The results are clearly, logically, and comprehensively presented, allowing the reader to readily follow the findings and their interpretation. The tables and figures are of good quality, are appropriately labeled, and effectively support the presentation of the results. Overall, no additional revisions to the Results section are required for acceptance.

**********

**Conclusions**

-Are the conclusions supported by the data presented?

-Are the limitations of analysis clearly described?

-Do the authors discuss how these data can be helpful to advance our understanding of the topic under study?

-Is public health relevance addressed?

Reviewer #1: The conclusions are well supported by the data presented and are consistent with the study findings. The limitations of the analysis are appropriately acknowledged and discussed, providing a balanced interpretation of the results. The authors clearly explain how their findings contribute to advancing the current understanding of the topic, and the public health relevance of the study is adequately addressed. Overall, the Conclusions section is appropriate and does not require further revisions for acceptance.

**********

**Editorial and Data Presentation Modifications?**

Use this section for editorial suggestions as well as relatively minor modifications of existing data that would enhance clarity. If the only modifications needed are minor and/or editorial, you may wish to recommend “Minor Revision” or “Accept”.

Reviewer #1: Accept

**********

**Summary and General Comments**

Use this section to provide overall comments, discuss strengths/weaknesses of the study, novelty, significance, general execution and scholarship. You may also include additional comments for the author, including concerns about dual publication, research ethics, or publication ethics. If requesting major revision, please articulate the new experiments that are needed.

Reviewer #1: This manuscript presents a well-conducted multicenter observational study describing the clinical and epidemiological characteristics of 590 hospitalized adults with laboratory-confirmed dengue during the 2025 outbreak in Bangladesh. The topic is timely and of considerable public health importance, given the increasing burden of dengue and its evolving epidemiological profile in the region. The multicenter design, the relatively large sample size, and the use of standardized data collection methods are important strengths that enhance the robustness and generalizability of the findings. The manuscript is well organized, the objectives are clearly stated, and the results are presented in a logical and coherent manner. Although some limitations inherent to the observational design remain and certain aspects could still be expanded in the discussion, these issues do not compromise the validity or interpretation of the findings. The revisions implemented by the authors have satisfactorily addressed the reviewers' previous comments and have substantially improved the quality and clarity of the manuscript. Overall, this study provides valuable data that contribute to the current understanding of hospitalized dengue cases and should be of interest to clinicians, epidemiologists, and public health professionals. I believe the manuscript is suitable for publication in its current form.

**********

PLOS authors have the option to publish the peer review history of their article (what does this mean?). If published, this will include your full peer review and any attached files.

If you choose “no”, your identity will remain anonymous but your review may still be made public.

**Do you want your identity to be public for this peer review?**  For information about this choice, including consent withdrawal, please see our Privacy Policy.

Reviewer #1: No

# Editor report: Acceptance letter

**DOI:** 10.1371/journal.pntd.0014661.r004

**Authors:** Eric Mossel, Sujatha Sunil

Dear Dr Tabassum,

We are delighted to inform you that your manuscript, "Clinical and Epidemiological Characteristics of the 2025 Dengue Outbreak in Bangladesh," has been formally accepted for publication in PLOS Neglected Tropical Diseases.

We have now passed your article onto the PLOS Production Department who will complete the rest of the publication process. All authors will receive a confirmation email upon publication.

The corresponding author will soon be receiving a typeset proof for review, to ensure errors have not been introduced during production. Please review the PDF proof of your manuscript carefully, as this is the last chance to correct any scientific or type-setting errors. Please note that major changes, or those which affect the scientific understanding of the work, will likely cause delays to the publication date of your manuscript. Note: Proofs for Front Matter articles (Editorial, Viewpoint, Symposium, Review, etc...) are generated on a different schedule and may not be made available as quickly.

Soon after your final files are uploaded, the early version of your manuscript will be published online unless you opted out of this process. The date of the early version will be your article's publication date. The final article will be published to the same URL, and all versions of the paper will be accessible to readers.

For Research Articles, you will receive an invoice from PLOS for your publication fee after your manuscript has reached the completed accept phase. If you receive an email requesting payment before acceptance or for any other service, this may be a phishing scheme. Learn how to identify phishing emails and protect your accounts at https://explore.plos.org/phishing.

Thank you again for supporting open-access publishing; we are looking forward to publishing your work in PLOS Neglected Tropical Diseases.

Best regards,

Shaden Kamhawi

co-Editor-in-Chief

PLOS Neglected Tropical Diseases

Paul Brindley

co-Editor-in-Chief

PLOS Neglected Tropical Diseases
